# Decoding Clot Waveform Analysis: Toward Better Understanding and Harmonization

**DOI:** 10.1055/a-2778-9810

**Published:** 2026-01-21

**Authors:** Jing Yuan Tan, Marvin Raden Torres De Guzman, Wan Hui Wong, Chi Kiat Yeo, Guan Hao Goh, Heng Joo Ng, Chuen Wen Tan

**Affiliations:** 1 Department of Haematology, Singapore General Hospital, Singapore

**Keywords:** coagulation, standardization, hemostasis, clot waveform analysis

## Abstract

Clot waveform analysis (CWA) extends routine coagulation assays (activated partial thromboplastin time [aPTT] and prothrombin time [PT]) by incorporating continuous optical monitoring to generate kinetic profiles of clot formation. This method provides both qualitative and quantitative information on hemostasis, with increasing evidence for its clinical utility in detecting factor deficiencies and characterizing thrombotic and bleeding disorders. Despite the growing body of evidence, translation of CWA into routine clinical practice remains limited.

This review identifies three principal barriers: (1) variability arising from differences in optical detection methods (absorbance vs. transmittance), (2) interreagent variation even within the same analyzer platform, and (3) lack of a clear distinction between standard CWA, performed with commercially available reagents, and modified CWA, incorporating in-house adjustments. To address these challenges, we encourage adopting distinct nomenclature for detection modalities (CWA-A; A for absorbance and CWA-T; T for transmittance), establishing standardized reporting requirements including reagent and platform details, and establishing quality assurance frameworks for CWA.

Standardization of terminology and reporting will enhance reproducibility, enable cross-study comparisons, and accelerate the clinical translation of CWA from the laboratory bench to the bedside.

Clot waveform analysis (CWA) has emerged as a promising investigational laboratory adjunct with potential clinical utility in the field of hemostasis and thrombosis, pending prospective validation studies. 1 Conceptually, it is an extension of the routine coagulation assays, including activated partial thromboplastin time (aPTT) and prothrombin time (PT), incorporating continuous optical monitoring of clot formation to generate kinetic profiles of coagulation. This approach provides a dynamic, quantitative assessment of an individual's coagulation status. The principles of CWA have been clearly described, most notably by Nogami in 2023, and its potential clinical applications have been extensively explored over the past decade. 2 Reported uses include (1) detection of factor deficiencies and severity assessment, (2) evaluation of anticoagulant effects, (3) characterization of bleeding and thrombotic disorders (e.g., venous thromboembolism), and (4) characterization of lupus anticoagulant (LA), among others. 3 4 5

Despite the growing number of studies published utilizing CWA, it has yet to achieve routine adoption in daily clinical practice. A key hindrance is the failure to standardize which hampers multicenter validation and high-quality evidence of clinical utility. We believe the underutilization also reflects substantial variability in CWA parameters generated across different coagulation analyzers, largely due to differences in clot detection methodology—absorbance versus transmittance—and in the reagents employed. To add to the confusion, various nomenclatures of CWA parameters have been reported in the literature and several in-house modifications of CWA methods and reagents have been employed. It would be important to refer such modifications to CWA as “modified-CWA” and should be specified in reporting to avoid confusion. 6 7 8 9 10 CWA should be reserved for the standard, unmodified CWA. Focusing only on standard CWA-enabled platforms with standard commercially available reagents, this review aims to provide clarity in this space. We believe such standard CWA are closest to routine clinical translation. In this review, we first provide a concise overview of CWA principles, followed by a comparison of outputs between different analyzer platforms and a consolidated summary of reagents and analyzer combinations used in published studies of CWA's clinical utility. Our objective is to highlight the heterogeneity in the existing literature and to advocate for the clear separation of CWA generated by different detection modalities. Such distinction would facilitate standardization, enable more meaningful clinical validation of published findings, and ultimately support the translation of CWA from the laboratory to the bedside.

## Clot Waveform Analysis

Automation of routine coagulation assays, such as PT and aPTT, has advanced rapidly over recent decades, with optical measurement of clot formation now widely adopted in diagnostic coagulation analyzers. Traditionally, these assays have been used to determine only the final clotting time, providing a one-dimensional snapshot of the coagulation process. However, growing evidence indicates that real-time analysis of the optical signal reveals dynamic changes in clot formation kinetics, offering a more comprehensive view of hemostasis.

**Fig. 1:**
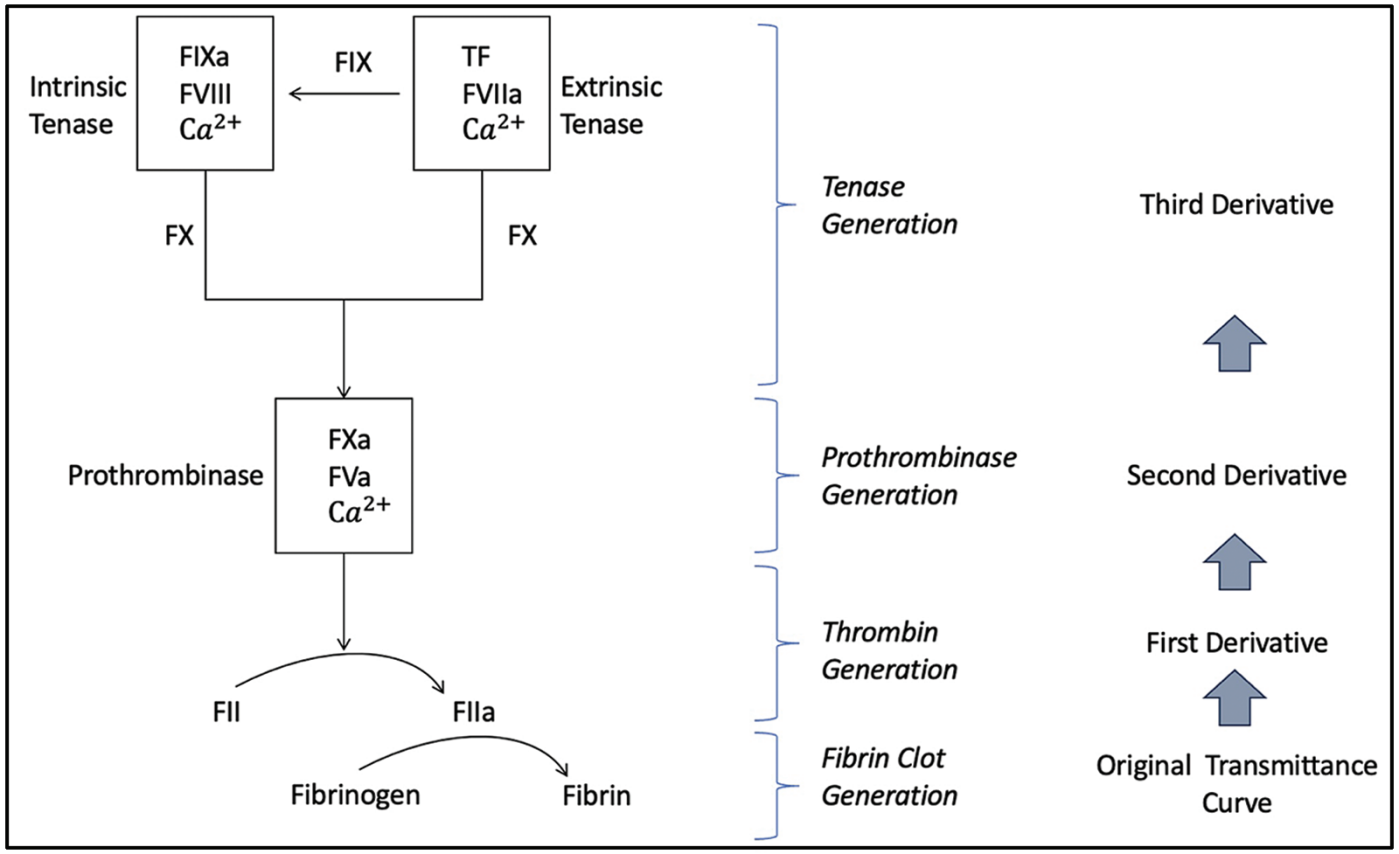
Diagrammatic representation of coagulation cascade events in relation to CWA curves. CWA, clot waveform analysis.

In platelet-poor plasma, hemostasis comprises three interconnected stages: (1) activation of the coagulation cascade via intrinsic or extrinsic pathways, (2) the thrombin burst, reflecting the positive feedback amplification of thrombin generation, and (3) fibrin formation through the action of activated clotting factors on platelet phospholipids. 11 Continuous optical monitoring captures changes in light transmission or absorbance during fibrin generation, generating a coagulation waveform that can be interrogated for both qualitative features and quantitative parameters—an approach now known as CWA. CWA involves processing the raw optical signal, typically through successive differentiation of the coagulation reaction curve, to derive key kinetic parameters: maximum velocity, maximum acceleration, and maximum deceleration. These parameters are obtained from first and second derivative curves. CWA allows an indirect assessment of various activation events that occur along the coagulation cascade, i.e., tenase complex activates factor X for the generation of prothrombinase complex, which in turn covert prothrombin to thrombin, which in turn converts fibrinogen to fibrin clot. Fig. 1 , in a crude manner, reflects these activation events in relation to CWA. The original transmittance curve reflects the rate of fibrin clot formation. The first derivative reflects thrombin generation activity. The second derivative reflects generation of prothrombinase and third derivative reflects generation of tenase complex. A German group has used advanced mathematical models to prove this relationship conceptually and there are indirect correlative data available. 12 13

## Quantitative and Qualitative Clot Waveform Analysis Parameters

Quantitative parameters are derived from the derivative curves generated by the analyzer. These parameters are reported in different units and formats depending on whether the analyzer records changes in light transmittance or absorbance. In the transmittance system, which is the most reported in the literature, maximum velocity is denoted as Min1, maximum acceleration as Min2, and maximum deceleration as Max2. In contrast, the absorbance system follows an opposite pattern, where maximum velocity, maximum acceleration, and maximum deceleration are referred to as Max1, Max2, and Min2, respectively. 14

The nomenclature and reporting of CWA parameters vary across studies, leading to confusion and hindering meaningful comparisons between published results. In addition, the raw transmittance or absorbance curve is strongly influenced by fibrinogen concentration, resulting in substantial variability in CWA parameters. To address this issue, Sysmex analyzers introduced the adjusted CWA, which mathematically compensates for the effect of fibrinogen on the clotting reaction curve. 15 However, further ambiguity has arisen because some studies have modified aPTT testing by incorporating alternative reagents or activators, generating what are essentially modified CWA parameters, but reporting them under the term “adjusted CWA.” 6 16 Such usage blurs the distinction, as these results are not mathematically derived in the same way as the conventional adjusted CWA. We therefore propose standardizing the terminology: “adjusted CWA” should refer exclusively to postanalytical fibrinogen adjustment, whereas “modified CWA” should be reserved for analyses that incorporate analytical modifications. Finally, we are aware of studies that have studied other points/parameters on the first and second derivative curves, such as “half of first derivative peak height” or “first derivative peak width;” the reporting of these “other” parameters should be clearly defined and best demonstrated with a diagrammatic representation. 10 17

**Fig. 2:**
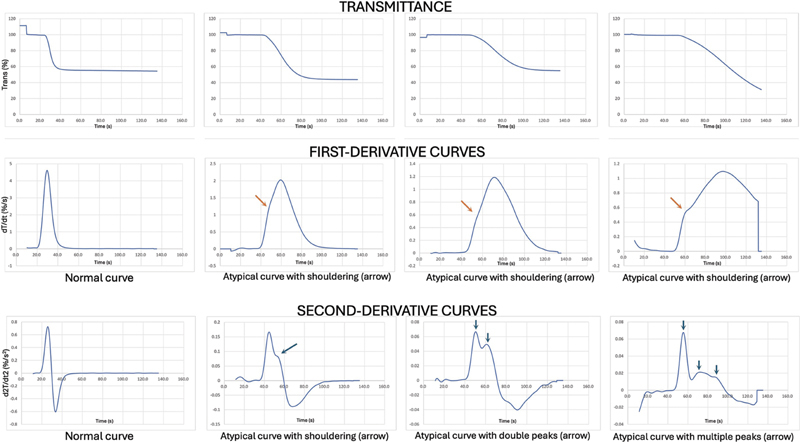
Patterns of atypical CWA curves with suggested nomenclature reporting. CWA, clot waveform analysis.

Qualitative CWA parameters, particularly the overall pattern of the clotting curve, have also attracted considerable research interest for their clinical applications in thrombotic and hemostatic disorders. The study of the CWA curve was first described in the late 1990s, when Toh and colleagues demonstrated the diagnostic utility of a characteristic biphasic waveform on the original transmittance curve in identifying patients with early disseminated intravascular coagulation (DIC). 18 This waveform was subsequently elucidated to arise from a calcium-dependent interaction between C-reactive protein and very-low-density lipoprotein, which produced a reproducible alteration in light transmittance during clot formation. 19 20 21 This biphasic waveform, however, was observed only on the MDA-180 platform, which is no longer commercially available. Despite this, the term “biphasic waveform” has continued to appear in the literature and is sometimes incorrectly applied to the double-peak patterns observed in the first- or second-derivative curves produced by ACL-TOP (Werfen) or CS/CN (Sysmex) analyzers. The biphasic waveform originally described by Toh et al. reflected progressive changes in the slope of the transmittance curve, with greater steepness correlating with clinical deterioration, and importantly, did not involve a double-peak configuration. There has been considerable differences in reporting of CWA waveform in both the first and second derivative curves including—“biphasic pattern of second derivative curve,” “dicrotic notch,” “shoulder type abnormal curve,” “biphasic-type curve,” “biphasic plot associated with double peak,” “biphasic waveform,” “atypical biphasic first derivative peak,” and “atypical second derivative shoulder peak.” 17 22 23 24 25 26 27 While all articles had diagrammatic representation to define the waveform nomenclature, for standardization and ease of comparison, we suggest reporting atypical clot waveform curves in the following manner (1) atypical; (2) first or second derivative curve; (3) shouldering vs double peak or multiple peaks. Diagrammatic representation continues to be important and we list examples below ( Fig. 2 ). This manner of reporting will eliminate ambiguity and avoid confusion.

## Comparing Clot Waveform Analysis between Analyzers and Reagents

Table 1 summarizes the CWA nomenclature, from existing available literature, reported for each platform. We are aware of newer coagulation analyzers from Sekisui that incorporate CWA algorithms based on optical absorbance and scattering method; 28 however, to date, no published reports in English have described their CWA parameters and clinical utility based on standard unmodified reagents.

**Table 1:** Comparison of two commonly used coagulation analyzers with clot waveform analysis output

	Analyzer 1	Analyzer 2
Company	Sysmex	Werfen
Analyzer	CS/CN-Series	ACL TOP family series
Optical method	Transmittance	Absorbance
Wavelength	340 nm 405 nm 575 nm 660 nm (main wavelength for aPTT/PT) 800 nm	405 nm 671 nm (main wavelength for aPTT/PT)
Clot time	Seconds	Seconds
Time to maximum velocity	Time to min1 Tmin1 Time to 1 ^st^ derivative peak (DP) 1 ^st^ derivative peak time	1 ^st^ Derivative peak time (DPT) Tmax1 Peak time velocity
Maximum velocity	Min1 |min1| a *Max1* “Ad” added if adjusted values are used	Max1 First derivative peak (first-DP) First derivative peak max First derivative peak height (DPH) First derivative height Velocity peak time Peak velocity Height velocity Height of 1 ^st^ DP Vmax (maximum coagulation velocity) ^c^ No equivalent adjusted value available
Units	%/sec	dmAbs/dt
Time to maximum acceleration	Time to min2 Tmin2 Time to 2 ^nd^ derivative peak (DP) 2 ^nd^ derivative peak time	2 ^nd^ derivative peak time (DPT) Tmax2 Acceleration peak time Peak time acceleration
Maximum acceleration	min2 |min2| a * Max _p_ 2 (p for positive) * “Ad” added if adjusted values are used	Max2 Second derivative peak (second-DP) Second derivative peak max Second derivative first peak height (DPH) Second derivative peak (+) Height of 2 ^nd^ DP1 Peak acceleration Amax (maximum coagulation acceleration) ^c^ No equivalent adjusted value available
Units	%/sec ^2^	mAbs/dt ^2^
Time to maximum deceleration	Time to max2 Tmax2	Deceleration peak time
Maximum deceleration	Max2 |max2| a Max _n_ 2 *(n for negative)* “Ad” added if adjusted values are used	Min2 Second derivative peak min Second derivative second peak height Second derivative peak (-) Height of 2 ^nd^ DP2 Dmax (maximum coagulation deceleration) ^c^ No equivalent adjusted value available
Units	%/sec ^2^	mAbs/dt ^2^
Difference in height	dH Δ change	Delta Absorbance Delta change
Units	%	mAbs
Additional parameters reported b		Width of first derivative peak Width of second derivative peak Area under curve (AUC)

Abbreviations: aPTT, activated partial thromboplastin time; PT, prothrombin time.

a Minority reporting in the literature that goes against the conventional nomenclatures.

b These parameters are not automatically calculated and made available on the analyzers. These are manually calculated and derived values.

**Fig. 3:**
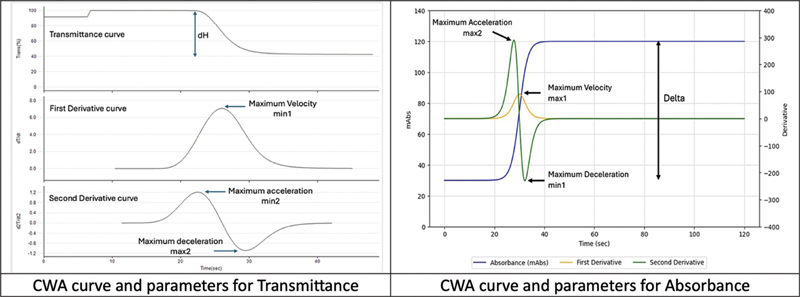
Comparison of CWA curves between transmittance and absorbance optical methods. CWA, clot waveform analysis.

As demonstrated in Fig. 3 and Table 1 , CWA values between two analyzers differ markedly—often by a factor of 10 to 100—primarily due to differences in the underlying optical detection methods. Furthermore, even within a single analyzer type, the reported nomenclature can vary. For example, the Sysmex platform has been described in the literature using terms such as *min1* , *|min1|* , and *ad|min1|* . At our center, we previously used the Sysmex CS-2500 and more recently transitioned to the Sysmex CN-3000. On both systems, the generated CWA data are reported as absolute values under the headings Max Velocity, Max Acceleration, and Max Deceleration, with corresponding adjusted values also provided (Max Velocity (adjusted), Max Acceleration (adjusted), Max Deceleration (adjusted)). Based on current published literature, placing *min1* , *min2* , or *max* within vertical bars (e.g., *|min1|* ) denotes the absolute value of the parameter.

To provide some clarity on the confusing nomenclatures, it helps to understand the underlying principle and history of the two main CWA methods. The use of min1 and the use of “absolute value” to denote maximum velocity is historical, as CS/CN (Sysmex) CWA is based on transmittance method and the direction of change is negative. In contrast, ACL TOP (Werfen) is using light absorbance, and the direction of change is positive and naturally, max1 denotes maximum velocity. At present, there is no standardization in the reporting of CWA parameters. Clinicians interpreting published data—particularly when aiming to validate or translate reported findings into their own practice—should be aware of these nomenclature differences and consider them carefully when comparing results across studies or platforms. We advocate users to report the CWA parameters according to nomenclature used by the manufacturers: CS/CN (Sysmex) analyzers—min1, min2, and max2 for max velocity, max acceleration and max deceleration, respectively; ACL TOP (Werfen) analyzers—max1, max2, and min2 max velocity, max acceleration and max deceleration, respectively.

Further heterogeneity arises when different reagents are used on the same analyzer. Table 2 shows the various reagents that are commonly used with the respective analyzer. Several studies have studied the differences in CWA between reagents. In a multicenter study, Wong et al. compared CWA parameters obtained with three reagents—Pathromtin SL, Actin FS, and Actin FSL—on the same Sysmex CS analyzer. Comparable CWA parameters were demonstrated across centers and reagent lots when the same reagents were being used. However, significant interreagent differences were observed. 29 Similarly, Wada et al. assessed the hemostatic performance of 10 different Factor VIII concentrates using CWA with 12 different aPTT reagents on the same ACL TOP analyzer and likewise found substantial variability in CWA parameters. 30

**Table 2:** Various reagents that are commonly used with respective analysers

Analyzer	aPTT reagent	PT reagent	Thrombin time
SYSMEX CS/CN Series	Pathromtin SL— *Silica* Actin FS— *Ellagic* Actin FSL— *Ellagic* Thrombocheck aPTT-SLA— *Silica* Thrombocheck aPTT— *Ellagic*	Thromborel S Innovin	Thrombin reagent Dade thrombin time Dade fibrinogen
ACL TOP Family series	HemosIL SynthASil— *Silica* HemosIL SynthAFax— *Ellagic* HemosIL Liquid aPTT— *Ellagic* HemosIL aPTT-SP— *Kaolin* HemosIL aPTT-SP— *Ellagic*	HemosIL RecombiPlasTin 2G HemosIL PT-Fib HS Plus HemosIL ReadiPlastTin	HemosIL thrombin Time HemosIL Fibrinogen-C XL

Abbreviations: aPTT, activated partial thromboplastin time; PT, prothrombin time.

By contrast, no published studies have directly compared aPTT-based CWA across different analyzers using the same reagent. Wada et al. evaluated the performance of various aPTT reagents in reference plasma using the ACL TOP analyzer, diluting reference plasma with Factor VIII-deficient plasma and recording the corresponding aPTT-CWA values. 30 At our center, we conducted a similar evaluation on the Sysmex CN-3000 (unpublished data) using a comparable dilution approach across a range of FVIII concentrations. In both their study and ours, reagents such as Actin-FSL, Actin-FS, and PSL were included, allowing for indirect comparison. Table 3 shows this comparison and highlights the significant differences in the reported CWA parameters.

**Table 3:** Comparison of activated partial thromboplastin time-clot waveform analysis data between ACL-TOP and CN 3000 analyzer using similar reagents

		ACL-TOP			CN-3000		
FVIII [conc.]	Parameter (ACL TOP/CN 3500)	Actin-FS	Actin-FSL	PSL	Actin-FS	Actin-FSL	PSL
0.5 IU/mL	1 ^st^ DPT (s)/time to min1	37.1 (37.0–37.3)	35.5 (35.2–35.7)	45.2 (45.2–45.5)	32.9 (32.9–33.0)	30.8 (30.7–30.8)	38.6 (38.2–38.9)
	1 ^st^ DPH a /Min1	264 (264–265)	250 (247–253)	197 (197–199)	2.996 (2.968–3.024)	3.509 (3.501–3.514)	1.95 (1.942–1.957)
0.1 IU/mL	1 ^st^ DPT (s)/Time to min1	60.8 (60.7–60.8)	54.5 (54.0–54.7)	73.4 (73.1–73.9)	45.5 (45.4–45.7)	40.4 (40.4–40.5)	48.3 (48.1–48.5)
	1 ^st^ DPH a /Min1	176 (174–176)	167 (166–168)	130 (128–130)	2.182 (2.169–2.202)	2.744 (2.731–2.760)	1.759 (1.75–1.775)
0.05 IU/mL	1 ^st^ DPT (s)/Time to min1	71.5 (71.4–72.0)	63.0 (62.8–63.3)	91.0 (90.4–92.0)	51.7 (51.6–51.8)	45.6 (45.5–45.8)	53.8 (53.7–54.0)
	1 ^st^ DPH a /Min1	145 (143–145)	139 (138–140)	108 (105–109)	1.875 (1.867–1.887)	2.36 (2.344–2.385)	1.591 (1.587–1.596)
0.01 IU/mL	1 ^st^ DPT (s)/time to min1	99.0 (98.7–99.2)	86.1 (85.3–86.3)	134 (134–135)	66.3 (66.2–66.4)	59.4 (59.0–58.8)	67.8 (67.7–67.9)
	1 ^st^ DPH a /Min1	90.4 (89.0–91.3)	91.8 (91.5–95.2)	71.7 (68.8–72.4)	1.278 (1.262– 1.298)	1.606 (1.593–1.615)	1.142 (1.136– 1.15)

Abbreviations: DPH, derivative peak height; DPT, derivative peak time; PSL, Pathromtin SL.

a Values for ACL-TOP are in (mAbs); values for CN 3500 are in (%/s).

In view of our recent transition from the Sysmex CS2500 to the Sysmex CN3000 coagulation analyzer, we also compared Actin FSL reagent on both platforms, we compared CWA parameters and observed an excellent level of agreement, with correlation coefficients exceeding 0.99 for all parameters. 31 These findings highlight the consistency of detection methods across the two analyzer models from the same manufacturer and indicate that CWA parameters are transferable when identical reagents are employed under these conditions.

## Consolidated Review of Clot Waveform Analysis Studies

We conducted a systematized narrative review to consolidate studies evaluating the clinical applicability of standard CWA across various hemostatic and thrombotic conditions. Our main aim was to summarize evidence derived from standard CWA performed on routine coagulation analyzers using commercially available reagents. As highlighted above, analyzer type and reagent composition significantly influence CWA parameters. Presenting the analyzer–reagent combinations used in each study enables more meaningful indirect cross-study comparisons.

**Fig. 4:**
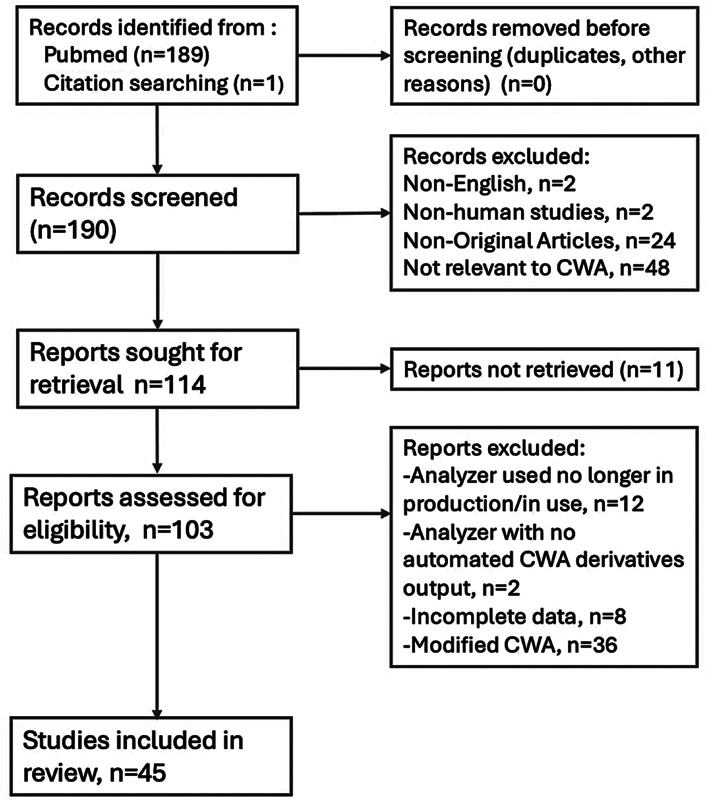
PRISMA flow chart for CWA studies. CWA, clot waveform analysis; PRISMA, Preferred Reporting Items for Systematic reviews and Meta-Analyses.

A structured PubMed search was performed from inception to June 18, 2025 using the keyword “clot waveform,” which yielded 189 records. A secondary search using “clot” AND “waveform” retrieved fewer articles. An additional record was obtained from citation searching from identified records. Screening and selection followed PRISMA (Preferred Reporting Items for Systematic reviews and Meta-Analyses)-informed methods: two independent reviewers evaluated titles and abstracts, with full texts retrieved for potentially eligible studies. We excluded non-English articles, nonhuman studies, nonoriginal publications (letters, conference abstracts lacking full text, protocols, and reviews), and studies unrelated to CWA. Additional exclusions included studies using obsolete analyzers, platforms without automated derivative outputs, incomplete methodological details (e.g., unspecified reagents), or in-house modified assays (“modified CWA”). Discrepancies were resolved by discussion with a third reviewer. Owing to substantial methodological heterogeneity, a quantitative synthesis was not feasible; therefore, we performed a structured narrative synthesis ( Table 4 ) and present the study selection process using a PRISMA-style flow diagram ( Fig. 4 ). A final total of 45 studies were included.

**Table 4:** Summary of studies on clot waveform analysis in various thrombotic and hemostatic conditions

Reference	Conditions	Reagent	Analyzer	Key CWA findings
Hypercoagulable states/thrombotic disorders				
32	COVID-19	Thrombocheck aPTT-SLA	CS-5100	CWA results showed significantly higher, min1, min2, max2, and median delta change in COVID-19 patients, consistent with hypercoagulability and were more pronounced in critically ill patients
33	COVID-19	Actin FSL	CN-6000	CWA in severe COVID-19 patients showed elevated delta change, clot velocity (min1), clot acceleration (min2) and deceleration (max2). Mild COVID did not show increased CWA parameters
34	COVID-19	Actin FSL	CS-2100i	COVID-19 subjects needing supplementary oxygen support had elevated CWA parameters including min1 and min2
35	COVID-19	Actin FSL	CN-6000	Critically ill COVID patients showed high min1, min2, max2 and delta change
36	COVID-19	Actin FSL	CS-2100i CS-2500	Rise of CWA parameters precedes and coincides with COVID-19 severity and intensive care unit admission
37	COVID-19	HemosIL SynthASil	ACL TOP 700	COVID-19 subjects showed distinct coagulation irregularities. Abnormal clot waveform patterns were observed. Covid 19 patients had higher maximum first derivative peak and higher maximum second derivative peak
38	Lupus anticoagulant	Actin FSL	CS-2100i	Study demonstrated lower min1, min2, max2 and more prolonged CWA times in LA positive samples
25	Lupus anticoagulant	HemosIL aPTT-SP	ACL-TOP	CWA may explain the significant atypical peak and D/A ratio extension in patients with LA-positive APS
39	Venous thromboembolism (VTE)	Actin FSL	CS-2100i	Higher CWA parameters were significantly associated with acute VTE
40	VTE	HemosIL SynthASil	ACL TOP 500	aPTT-CWA parameters were higher in hospitalized medical patients with high venous thromboembolic risk (scored via Padua Prediction Score [PPS]) vs. those with low VTE risk and healthy subjects. The second derivative peak showed significant association with a high PPS score
41	Acute myocardial infarction	Actin FSL	CS-2100i	Acute myocardial infarction Patients with acute myocardial infarction had higher CWA parameters. AMI patients with complications had higher min1 and min2 than AMI patients who had no complications
Hypocoagulable states/bleeding disorders				
42	Hemophilia A and B	HemosIL SynthASil	ACL-TOP 500	The width of acceleration 1 [+] peak differentiates between HA (noninhibitors) and HB samples. The second derivative [+] peak was lower in mild and moderate HA. The aPTT-CWA parameters may be supportive for the differentiation of hemophilia A and B including its severity and the existence of inhibitors
43	Hemophilia A	Dapttin (technoclone) Actin FSL Pathromtin SL Triniclot aPTT-SP	ACL Pro Elite	Significant variability in CWA parameters were demonstrated between reagents that were used in the aPTT assay for measuring factor VIII
13	Hemophilia A	HemosIL SynthASil	ACL TOP 500	CWA parameters showed a linear relationship to factor VIII levels over a very large concentration range and was able to discriminate patients with severe, moderate and mild hemophilia A
24	Hemophilia A Hemophilia A with emicizumab	Thrombocheck aPTT-SLA Thrombocheck aPTT Data-Fi HemosIL SynthASil	CN-6000	Results showed a significant difference among CWA parameters in various reagents among hemophilia A patients
44	Hemophilia A	Automate	CS-2000i	CWA parameters can be effective in distinguishing hemophilia A patients with clinically severe bleeding vs. nonsevere bleeding phenotype
45	Hemophilia A and B	Actin FS	CS-2500	Compared global hemostatic assays between hemophilia A and B using TEG, TGA, and CWA. No statistically significant difference between MIN2 of CWA in patients with hemophilia B with those of hemophilia A
46	Hemophilia A	Thrombocheck aPTT-SLA Actin FS Actin FSL	CS-2000i	Studied utility of CWA in differentiating hemophilia A (HA) patients with absent Factor VIII levels vs very low Factor VIII levels. CWA parameters were lower in HA with inhibitors compared to other groups. Designed a composite parameter slope-min1, that could be useful in evaluating very low/ absent levels of FVIII and the development of FVIII inhibitor
47	Hemophilia A Acquired hemophilia A	Thrombocheck aPTT-SLA	CS-2000i	CWA revealed different decreased maximal coagulation velocities across patients with mild/moderate hemophilia A, acquired hemophilia A and lupus anticoagulants
48	Lupus anticoagulant Heparin Direct oral anticoagulant Hepatic dysfunction factor deficiency with inhibitor	HemosIL aPTT-SP	ACL-TOP	Classification of aPTT prolongation using the initial-to-peak gradient, the ratio initial-to-intermediate velocity/intermediate-to-peak velocity, and the initial-to-peak area size is possible
49	Hemophilia A carriers	HemosIL-Synthasil	ACL TOP 350	Tmax 1 and Tmax 2 were significantly prolonged in hemophilia carriers compared to controls. Max 1 and Max 2 were significantly lower in carriers. Using ROC analysis, aPTT-CWA parameters cutoffs showed good sensitivity and specificity in discriminating between carriers and controls. When comparing bleeders and nonbleeders carriers, a significant difference was noted in Max 2, Min 2, Tmax 1 and Tmax 2
50	Fibrinogen disorders	Thrombocheck Fib(L)	CN- 6000	CWA-Clauss of the subjects showed a prolonged clotting time with a slower clotting rate than normal plasma and was able to identify qualitative fibrinogen abnormalities even when fibrinogen levels are upregulated
51	Fibrinogen disorders	Dade thrombin	CN- 6000	The FbAc/eAg ratio can serve as a cost-effective parameter for identifying dysfibrinogenemia FbAc—fibrinogen activity derived from Clauss fibrinogen assay eAg is derived from the maximum velocity value (min1) of the first derivative curve of the Class fibrinogen CWA
52	Fibrinogen disorders	Thrombocheck FibL Dade thrombin	CN-6000	Clauss-CWA offers a novel approach to detecting qualitative fibrinogen abnormalities. Authors proposed a cutoff of 0.65 for FbAc/eAg ratio, which has excellent sensitivity and specificity
53	Fibrinogen disorders	Thrombocheck Fib (L)	CN-6000	A novel parameter |min|c was obtained from Clauss-CWA first derivative curve and correlated well with fibrinogen antigen. Fibrinogen activity/|min1|c ratio could detect qualitative disorders
54	Fibrinogen disorders	Thrombocheck Fib (L)	CS-2400	Clauss/dH and Clauss/Min1 ratio were useful in screening dysfibrinogenemia patients. dH and min1 were derived from Clauss-CWA. dH—total difference in transmittance level for fibrinogen formation, and min1—derived from first derivative curve and an indicator of the maximum velocity of coagulation
55	Fibrinogen disorders	Thrombocheck Fib (L)	CS-5100	CWA of CFA could distinguish fibrinogen disorders using a combination of Fibrinogen Activity (Ac)/Min1 and Ac/eAg values and represents a novel screening test for fibrinogen disorders
56	Disseminated intravascular coagulopathy	Actin FS	CS-2500S	Studied aPTT-CWA in assessing Disseminated intravascular coagulopathy in critically ill children. Min2 of less than 0.36%/s ^2^ was identified as a prognostic factor for bleeding in children with DIC
57	Snakebite-coagulopathy	HemosIL-Synthasil	ACL TOP 300	CWA may reveal disorders of clotting in snakebite victims before standard tests such as WBCT or aPTT becomes abnormal. This study reported atypical biphasic waveform seen in venom-induced consumptive coagulopathy
58	Lupus anticoagulant Acquired hemophilia A	Thrombocheck aPTT-SLA	CS-2400	In terms of CWA, study showed that the parameter adjusted|min 1| can be useful for discriminating AHA from LA, because it is markedly lower in AHA while it is only marginally low in LA. However, this is caveated that the Ad|min1| of CWA in some high-titer LA cases can be comparable to that of AHA, making the distinction difficult
17	Hemophilia A and B Thrombosis Hepatic dysfunction	HemosIL SynthASil	ACL TOP 500	Studied CWA in various disorders including hemophilia A and B, thrombosis and hepatic dysfunction. Reported biphasic pattern of first and second derivative curve in hemophilia patients. Patients with hepatic dysfunction showed prolonged First DC and Second DC peak time. Patients with thrombosis showed marked elevation in first DC
Anticoagulants				
59	Anticoagulants	Actin Actin FSL Coagpia aPTT-N HemosIL SynthASil	CS-5100 CN-6000	CWA parameters were able to differentiate between antithrombin dependent and antithrombin independent anticoagulants
60	Warfarin	Innovin Actin FSL	CS2100i	Evaluated aPTT-CWA and PT-CWA parameters correlation with TGA parameters in warfarin treated patient. aPTT-CWA demonstrated better correlation with TGA parameters than PT-CWA
15	DOACs	RecombiPlasTin 2G Revohem aPTT SLA	CS-5100 CN-6000	Adjusted PT-CWA parameters exhibited dose-dependent decrease with increasing DOAC concentrations. Both nonadjusted and adjusted aPTT-CWA parameters showed dose-dependent decrease with increasing DOAC concentrations
61	DOACs	HemosIL SynthasIL ReadiPlasTin	ACL TOP 700	PT-CWA and the FibWave parameters were better correlated with edoxaban levels than aPTT-CWA parameters
62	Direct thrombin inhibitors	Dade Actin FSL Coagpia aPTT-N HemosIL SynthASil	CS-5100	Blockade of thrombin-positive feedback by bivalent direct thrombin inhibitors, hirudin, and bivalirudin, was demonstrated by CWA. The dose–dependent curves in CWA were different between hirudin and bivalirudin. Distinct features of hirudin and bivalirudin likely related to the different reversibility were revealed by CWA
Miscellaneous				
29	Reagent differences	Pathromtin SL Actin FS Actin FSL	CS 5100 CS 2100i CS 2500	Multi-center study. Different reagents were tested on the same analyser series using healthy controls. aPTT-based CWA parameters were significantly different between reagents. References ranges were established in this study
63	Hemodilution effect	Thrombocheck aPTT-SLA	CS-5100	Study looked at hemodilution effect on CWA parameters. CWA parameters (Min1, Min2, Max2) showed a linear, dilution-dependent decrease closely following the drop in clotting factors with dilution
64	CWA in infections	Actin FSL	CS2100i	CWA parameters demonstrated positive correlation with CRP levels. Different infections had different aPTT-CWA parameters. Infections in this study included bacterial infections, dengue, and respiratory viral infections
65	Hepatobiliary surgery	aPTT-SP	ACL TOP	Assessed utility of aPTT-CWA in predicting risk of bleeding after major hepatobiliary surgery. aPTT-CWA was more sensitive than conventional aPTT assay in detecting postoperative hemostatic changes after major HBP surgery. Lower 1st and 2nd DP heights were possible predictors for major bleeding risk
66	Liver cirrhosis	RecombiPlasTin 2G	ACL TOP 500 CTS	Assessed the role of PT-CWA in predicting bleeding risk in liver cirrhotic patients. The combination of area of parabolic segment of 1 ^st^ derivative curve of PT-CWA with PT ratio predicted bleeding risk with an AUC of 0.72 (95% CI: 0.67–0.77)
67	Acute promyelocytic leukemia	Synthasil RecombiPlasTin 2G	ACL TOP 350	Assessed aPTT-CWA and PT-CWA in patients with APML. Both aPTT-CWA and PT-CWA parameters were significantly lower in APML patients with DIC and hypofibrinogenemia. CWA parameters showed a strong and significant correlation with fibrinogen levels
68	Chronic spontaneous urticaria (CSU)	HemosIL SynthASil	ACL TOP 550	The 2nd Derivative of aPTT showed lower values in CSU than those of the HC which may indicate a low grade DIC
69	Liver cirrhosis	Silica liquid aPTT	ACL TOP 500 CTS	Assessed the role of aPTT-CWA in liver cirrhotic patients. CWA parameters were significantly lower in those patients who presented a clinical history of bleeding in comparison with patients who did not. CWA parameters were higher in cirrhotic patients with a higher Padua Prediction Score (PPS) for venous thromboembolism
70	Lupus anticoagulant hypoprothrombinemia syndrome	Thrombocheck aPTT-SLA	CS 2400i	CWA appeared to underestimate the true coagulation capacity. Because the CWA trigger reagents contain synthetic phospholipids, the results can be markedly influenced by the presence of lupus anticoagulant

Abbreviations: aPTT, activated partial thromboplastin time; CI, confidence interval; COVID-19, coronavirus disease 2019; CWA, clot waveform analysis; PT, prothrombin time.

Of the 45 articles included in this consolidated review, 16 studies utilized the ACL TOP (Instrumentation Laboratory/Werfen) series of instruments while 29 studies utilized Sysmex (Sysmex Corporation, Kobe, Japan) automated analyzer. Numerous different reagents were used in the studies. Specific reagents are generally paired with specific coagulation analyzer as was highlighted in Table 2 . The clinical conditions in which CWA were studied were categorized into four main groups: (1) CWA in Thrombotic/Pre-thrombotic disorders, (2) CWA in Bleeding Disorders, (3) CWA in Drugs/Reagents, and (4) CWA in Miscellaneous conditions. Table 4 summarizes the relevant articles.

### Study Quality and Risk of Bias Assessment

The overall methodological quality of the included studies was limited by their predominantly retrospective design. Of the 45 studies included, the majority were retrospective observational cohorts or single-center laboratory/analytical investigations. Only three studies 33 56 65 employed a prospective clinical design, and only one study 29 was a multicenter analytical evaluation. No interventional studies, randomized designs, or large prospective cohorts were identified.

Formal risk-of-bias tools commonly used for clinical intervention studies were not applicable due to substantial heterogeneity in study purpose, ranging from assay-validation projects and reagent/analyzer comparisons to mechanistic exploratory analyses. Across the evidence base, the principal potential sources of bias included selection bias inherent to retrospective sampling, heterogeneity in analyzer–reagent combinations, inconsistent reporting of assay conditions, and incomplete standardization of CWA methodology. These limitations reflect the developmental stage of CWA research and collectively underscore the need for harmonized reporting standards, multicenter validation studies, and well-designed prospective clinical investigations.

The clinical potential of CWA has been underscored in several recent review articles. These reviews often consider both standard and modified CWA together, which risks conflating two approaches with distinct roles. Standard CWA, performed on routine coagulation analyzers with widely available reagents, is the modality most immediately applicable to clinical practice. By contrast, modified CWA—though methodologically innovative—remains primarily a research tool for exploring hemostatic mechanisms. Distinguishing between these two is essential to clarify the translational trajectory of CWA. In our own work, we demonstrated that CWA parameters can be pooled and compared across institutions when measurements are performed on the same platform and reagent type. 29 Such reproducibility is a key prerequisite for broader clinical adoption and underscores the robustness of standard CWA under harmonized conditions.

The body of evidence as summarized above is based solely on standard CWA and while predominantly retrospective and heterogeneous with respect to reagents, nonetheless points to a consistent conclusion—i.e., elevated CWA parameters are generally associated with hypercoagulable states, whereas reduced parameters reflect hypocoagulability. Importantly, standard CWA yields information on hemostatic function that extends beyond conventional clotting times and shows moderate correlation with thrombin generation assays. 60 64 Finding actionable cutoff values would be an important next step in translating CWA into patient management. Understanding the interference on CWA by various factors in real life, such as LA, direct oral anticoagulant, factor deficiencies whether in isolation or in combination is also crucial to move this field forward. Meanwhile, CWA can be regarded as a methodological hemostatic assay with potential adjunctive value, particularly where serial measurements can be obtained.

## Toward Better Harmonization in Clot Waveform Analysis Use and Reporting

Over the past two decades, research into CWA has firmly established its role as a valuable adjunct to routine coagulation testing. Beyond a single clotting time, CWA yields a wealth of qualitative and quantitative data that can inform and assist clinical decision-making in both thrombotic and bleeding disorders. The key challenge now lies in translating this potential into consistent, bedside-relevant practice. We propose several steps that may help bridge the gap between laboratory measurement and clinical application:

### Establishing a Dual Nomenclature for Optical Detection Methods

As highlighted in our current review, reporting of CWA parameters varies greatly between reagents and analyzers, with the most fundamental difference being the optical detection method—absorbance versus transmittance. These modalities produce significantly different numerical values, making direct comparison and translation of results across platforms problematic. We therefore propose adopting two distinct nomenclature systems: CWA-A (absorbance-based) and CWA-T (transmittance-based). This classification would provide clarity, improve uniformity in subsequent studies, and ensure that translation and validation of results are modality-specific. Our proposal mirrors the approach used in viscoelastic testing, where rotational thromboelastometry and thromboelastography operate on the same underlying principle but employ different nomenclature to describe their parameters and both have different reference intervals and actionable points.

We propose that the minimum reporting standard to include the CWA optical method, the analyzer used with model number, the type of CWA, the reagents, the clot time, and CWA parameters derived along with corresponding units as described in Table 5 .

**Table 5:** Proposed minimum reporting standard for clot waveform analysis

CWA optical method	CWA-A (absorbance)	CWA-T (transmittance)
Analyzer (model number to be stated)	CS/CN-Series	ACL TOP family series
Type of CWA	PT-based, aPTT-based, fibrinogen-based, thrombin time (TT)-based	PT-based, aPTT-based, fibrinogen-based, TT-based
Reagents used, examples	Pathromtin SL, Actin FS/FSL	SynthASil, Liquid aPTT
Clot time	Reported in seconds	Reported in seconds
Time to maximum velocity	Tmin1 (s)	Tmax1 (s)
Maximum velocity (units)	min1 (%/sec)	max1 (dmAbs/dt)
Time to maximum acceleration	Tmin2 (s)	Tmax2 (s)
Maximum acceleration (units) a	min2 (%/sec ^2^ )	max2 (mAbs/dt ^2^ )
Time to maximum deceleration	Tmax2 (s)	Tmin2 (s)
Maximum deceleration (units) a	max2 (%/sec ^2^ )	min2 (mAbs/dt ^2^ )

Abbreviations: aPTT, activated partial thromboplastin time; CRP, C-reactive protein; CWA, clot waveform analysis; PT, prothrombin time.

a Prefix “Ad” to parameters when adjusted values are obtained directly from the coagulation analyser.

b If derived or custom parameters beyond those routinely reported by the coagulation analyser are included, these should be explicitly described in the methodology. The parameter labels or symbols used should be distinct from the standard nomenclature outlined above to avoid ambiguity.

c If any modifications are made to reagents or testing samples, this should be clearly stated in the methodology, and the prefix “Mod” should be used.

### Distinguishing Standard from Modified Clot Waveform Analysis for Clinical Translation

Reagent differences, in addition to analyzer methodology, remain a major contributor to interlaboratory and interstudy variability. Different coagulation activators, phospholipid content, and reagent formulation can substantially alter waveform characteristics and the parameters derived from them. Within this context, it is paramount to distinguish between standard CWA and modified CWA, as the two approaches serve distinct purposes.

Standard CWA refers to analyses performed with commercially available reagents on routine analyzers using established protocols. Because these reagents are derived from assays already standardized for PT and aPTT testing, results obtained on the same analytical platform are comparable and can be generalized across centers. In our most recent multicenter collaboration, reagent- and platform-specific reference intervals were developed, supporting the feasibility of wider clinical adoption and the establishment of decision thresholds. To facilitate clinical translation, the priority should be the standardization of reporting in the literature, thereby enabling cross-study comparisons, improving reproducibility, and promoting the integration of CWA into routine patient management.

By contrast, modified CWA encompasses approaches that involve in-house modifications of reagents, protocols, or measurement times. An example is the clot-fibrinolysis waveform analysis, which extends measurement beyond clot formation into the fibrinolytic phase. While these modifications expand the potential scope of CWA, they remain experimental and are not yet ready for clinical application. Their translation would require extensive validation and consensus building, a process that may take considerable time.

Taken together, both forms of CWA are valuable but serve different roles. Standard CWA represents the more immediate and accessible path to clinical translation, while modified CWA functions as a research platform to explore new capabilities. Recognizing this distinction is important when appraising existing literature to understand the clinical applicability of the published results.

### Establishing Quality Assurance Frameworks for Clot Waveform Analysis

The establishment of internal and external quality control systems will be essential to ensure reproducibility and comparability of CWA across laboratories. This should be readily achievable, as routine PT and aPTT testing already operates within robust internal quality control (IQC) and external quality assurance (EQA) frameworks. Because CWA is derived directly from these assays, quality control for CWA can largely leverage the same infrastructure, materials, and processes. In practice, this means that adapting existing PT/aPTT quality systems to include waveform-derived parameters would provide a straightforward pathway to implementation. Combined with standardized reporting conventions, such measures will form the foundation for reliable translation of CWA into clinical practice.

## Operational Feasibility and Implementation

The availability of CWA as a 24/7 test is largely dependent on the individual laboratory's operational schedule. CWA functionality is inbuilt within many modern coagulation analyzers; no dedicated software, hardware upgrade, or additional reagents for standard curve generation are required. Access to CWA parameters typically depends on the manufacturer granting user rights to export or visualize derivative data generated from the routine coagulation assays (e.g., aPTT, PT, fibrinogen, thrombin time).

Because CWA parameters are mathematically derived from the clotting curve of existing tests, there is no additional per-test cost apart from data extraction or postprocessing time. No extra maintenance or service requirements are imposed beyond those already stipulated for the base coagulation analyser. However, individual laboratories should continue participation in routine IQC and EQA programs for the underlying coagulation assays on which CWA is based.

Integration of CWA data into the laboratory information system would represent the ideal pathway for clinical translation, allowing longitudinal data capture and automated trend analysis. Such integration would require collaboration with instrument manufacturers and potentially customized middleware development. Continued research and generation of robust clinical validation data are therefore essential to support broader adoption and standardization of CWA reporting.

## Conclusion

CWA has evolved from an experimental concept to an investigational hemostatic assay with potential adjunctive value applied in the areas of detecting factor deficiencies, monitoring anticoagulant therapy, characterizing disorders such as myocardial infarction, venous thromboembolism events, and LA. Yet, despite growing applications in various clinical hemostatic settings, translation into daily clinical practice remains limited primarily due to the paucity of high-quality multicenter prospective validation studies to demonstrate clinical benefit—an effect of lack of CWA standardization.

Our review highlights three principal barriers: (1) variability in CWA parameters arising from fundamental differences in analyzer optical detection methods (absorbance vs transmittance), (2) substantial inter-reagent variation, even within the same analyzer platform, and (3) the lack of distinction between “standard” and “modified” CWA. These factors complicate cross-study comparisons, hinder standardization, and limit the development of universally applicable diagnostic thresholds.

To address these challenges, we propose: (1) adoption of distinct nomenclature systems to differentiate absorbance-based (CWA-A) from transmittance-based (CWA-T) parameters; (2) establishing standardized reporting requirements for CWA, including a minimum requirement to specify the reagents and analyzer platforms used, and to clearly distinguish between standard CWA (for clinical use) and modified CWA (for research use); (3) establishing quality assurance frameworks for CWA. Implementing these measures would improve reproducibility, facilitate interlaboratory validation, and ultimately support the integration of CWA into routine clinical decision-making. By moving toward a standardized, modality-specific approach, the field can accelerate the translation of CWA from the laboratory bench to the patient bedside.
